# You realise you are better when you want to live, want to go out, want to see people: Recovery as assemblage

**DOI:** 10.1177/00207640211019452

**Published:** 2021-05-21

**Authors:** Inger Beate Larsen, Jan Georg Friesinger, Monica Strømland, Alain Topor

**Affiliations:** 1 Department of Psychosocial Health, University of Agder, Grimstad, Norway; 2 Department of Social Work, Stockholm University, Stockholm, Sweden

**Keywords:** Recovery, mental health, assemblage, narratives

## Abstract

**Background:**

The lack of social and material perspectives in descriptions of recovery processes is almost common in recovery research.

**Aim:**

Consequently, we investigated recovery stories and how people with mental health and/or addiction challenges included social and material aspects in these stories.

**Method:**

We conducted focus group and individual interviews. We investigated how the participants narrated their stories and how they assembled places and people in their recovery stories.

**Results:**

We found that narratives of recovery became assemblages where humans and their environments co-exist and are interdependent.

**Conclusion:**

As such, narratives about recovery are about everyday assemblages of well-being into which stories of insecurity are interwoven, without a start or stop point.

## Background

In the middle of the 20th century, two simultaneous processes put recovery from severe mental health problems on the agenda. (1) A number of long-term follow-up studies were published that challenged the understanding of schizophrenias as long-term illnesses, and some of them stressed the socio-cultural aspects involved in recovery processes (Bleuler, 1978; Ciompi, 1980; Harding et al., 1987; Warner, 1985/2004; WHO, 1979). (2) Inspired by black and women’s liberation movements fighting for their civil rights, users of mental health services started to organise themselves (Davidson et al., 2010).

Paradoxically these societal aspects disappeared in the most widely accepted definition of recovery formulated by Anthony (1993). His definition focuses on recovery within an illness model as an individual process that is concerned solely with changes within the person, without considering their social and material context. This lack of social and material embeddedness in the description of recovery processes is common in recovery research and mental health policies (Leamy et al., 2011; Price-Robertson et al., 2016; Tew et al., 2012). However, there are some studies focusing onmaterial aspects of recovery which is covered in the work on ‘social exclusion’ (Boardman, 2011; Ramon, 2018). Boardman (2011) emphasis the connection between material resources like money, housing and other goods as vital for being a citizen and for the recovery process. He describes how people having mental health problems suffer from ‘material deprivation’ because they often live in poor neighbourhoods and poor housing conditions, resulting in social exclusion and non-recovery (Boardman, 2011). To specifically include the social and material perspective, Topor et al. (2020) suggested a social model to understand recovery:

Recovery is a deeply social, unique and shared process in which our living conditions, material surroundings, attitudes, values, feelings, skills, and/or roles are changing.

It is a way of living satisfying, hopeful, and contributing lives, together with others even though we may still experience distress, unusual experiences and troubled or troubling behaviour.^1^

Recovery involves engaging in new material and social contexts and in open dialogues where new understandings of the situation we find ourselves in are created as we move beyond a psycho-social-material crisis.

This model emphasises that recovery occurs with others at specific places, which are embedded in what is called therapeutic landscapes. As such therapeutic landscapes could be described with the concept of Gesler (1992, 2003) and Williams (1998, 2017), involving social, material and symbolic aspects that form experiences, beliefs and recovery narratives.

Therapeutic landscapes meant for people with mental health problems can be meeting places (Larsen & Topor, 2017), supported housing (Friesinger et al., 2019, 2020), inpatient settings (Wood et al., 2013) or places focusing on green environments with a connectedness to nature (Bell et al., 2014). They are meant to be places that help people to be included in society as citizens. Since these places are meant for a specific group of people, they might constantly remind the visitors that they differ from the people ‘out there’ (Larsen & Topor, 2017). However, Duff (2012) writes about enabling places that promote mental health recovery. In the lens of therapeutic landscapes, such enabling places can be understood as assemblages of health (Duff, 2014).

### Assemblage

To contextualise recovery processes, the concept of assemblage could provide an appropriate toolkit, because the concept includes both social and material aspects of peoples’ lives and might broaden the recovery perspective. Bennett (2010) shows how humans relate to the environment in powerful, material assemblages, meaning that the relationship between humans and non-humans should be read as a blending. As such an assemblage which might consist of people, bodies, materials (e.g. houses, furniture, objects), ideas, and practices, and is therefore characterised as socio-material (DeLanda, 2006, 2016). DeLanda (2006) highlights the heterogeneity of the interrelationships of the parts included in an assemblage and shows how objects and practices matter inside and beyond spaces of recovery. As such, a bench might reveal opportunities for friendships, because someone you like can come and sit beside you (Larsen et al., 2020), and this new friendship can lead you to other places and new people; or it can end. DeLanda (2006) underlines that assemblages have no significant start or stop point for their interrelations, and new relations within assemblages can easily emerge or disappear or change. Assemblages are therefore fluid arrangements that account for social complexity and are not static or passive constructions. In this way, assemblages are defined by their connections rather than their boundaries; by their routes rather than roots (Deleuze & Guattari, 1987).

As such, recovery might emerge through specific assemblages which affect people’s bodies and their lives (Andrews & Duff, 2019). A place understood as a specific assemblage allows us to understand the well-being or illnesses of people in their daily lives better than theories that are based on health dichotomies or functions that reduce the social complexity. For example, to recover from mental health problems (Leamy et al., 2011) might be seen as a journey of becoming well in different socio-material contexts (Larsen et al., 2020; Topor et al., 2011). A recovery story could be a story of taking a big step away from a troublesome past and seek for places where you experience well-being. It might be a story about inviting friends for a cup coffee at a nice coffee-shop or participating in a peer driven meeting place and thus different experiences far from the traditional patient role. These situated experiences could contribute to the development of a different sense of self. Duff (2014, chap. 4) describes assemblages of health as places that provide ‘social, affective and material resources’.

## Method

In this study, we asked people with service user experience within mental- and/or addiction services themselves about their paths to recovery.^2^ As we wanted to explore first-hand descriptions of lived experiences, we chose group interviews and in-dept interviews for data production. We tried to catch descriptions of the participants experiences rather than their analytical perspective of what happened. Further, we use the term’ data production’ because we, in line with Brinkmann and Kvale (2015), believe that qualitative data is not something existing ‘out there’ that you can simply ‘collect’. We acknowledge that the process of putting lived experiences into words, is influenced by the interaction between the different participants in the group interviews and the researcher, and between the participant and the researcher in the individual interviews. We were interested in their personal experiences related to both mental health and addiction services, and also related to their living situation in general. We wanted concrete and detailed descriptions of the experiences they spoke about, hence these qualitative methods are well suited.

The study gives knowledge about how the participants themselves narrate their complex experiences of recovery (Squire et al., 2013). With the help of assemblage theory, we will investigate the participants’ recovery stories, and how they included different (material) places and people of importance, to understand their lives and recovery processes.

## The data production

We conducted both focus group- and individual interviews to collect data. When planning the project, the idea was to start with focus groups and then have the possibility to ask individuals from the group to go deeper into topics we wanted to elaborate on. We planned to start with focus group interviews to explore our topic and let the participants reflect on each other’s narrations. This method is based on dialogue and the dynamics between the participants in the group, and the interviewer is more interested in listening to the conversation between the participants and the stories they tell each other, than asking them questions (Krueger & Casey, 2000).

Our idea to start with focus group interviews ended somewhat differently because some participants were eager to join a group, others wanted to talk pair to pair, and some wanted to meet us alone. Additionally, in one group the participants also wanted the leader of the meeting place to join the group, because, as they said, ‘she is one of us’. In this situation we decided that the different modes of getting access to users’ experience-based knowledge could be seen as complementary and increased the richness and depth of the experiences.

The different interview forms lasted between 60 and 90 minutes. They were audio-recorded and transcribed verbatim. The text length was 174 pages (Times New Roman/12).

## The participants

We recruited participants from different meeting places open to people with mental health- and/or addiction challenges in different Norwegian municipalities. We asked the managers of the places to ask the members to participate, and they were all sent a written information letter. For the study, we invited participants that considered themselves as recovered, not necessarily in the sense of clinical recovery, but in the sense of having got a better life.

**Figure 1.**
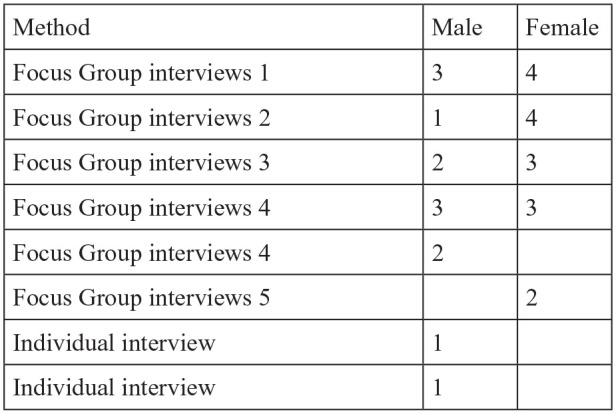
The different methods and participants.

Altogether, 29 persons participated; 16 women and 13 men (Figure 1).

## The interviewers

We both have researchers by profession and researchers by experiences (service user researchers) as members in our research group, and we decided that the two occupational parties would conduct focus group interviews 1, 2, 3 and 4. The same researcher by experience led the discussions, while the researcher by profession acted as an observer and a moderator. The two focus groups with the two participants were conducted of different researchers by profession and the two individual interviews were both conducted by the same researcher. Altogether we were four researchers by profession and one researcher by experience contributing to the data production.

## Ethical considerations

The Norwegian Centre for Research Data (NSD) accepted the project (number 535168). All the participants gave informed voluntary consent. It was important that they did not feel obligated to participate, therefore we asked the managers to ask them on our behalf. When contacting the managers, we emphasised that participation was voluntary. Regarding confidentiality, all participants are anonymous, and names are invented.

## The validity of this study

Participants’ experiences are depicted using their own descriptions of various situations related to what they considered as having promoted their recovery. In the focus group their personal stories are stories told in a group. The strength of the four largest focus groups might be that the members encouraged each other to be open about their experiences. The same was true for the two small focus groups. The participants in the two individual interviews were meeting the same researcher, and they both said that it was important for them to tell their stories not being afraid that sensitive information about themselves would be known to others. We might consider the different methods to supplement each other, because what you get when participants are discussing recovery is not necessarily the same as what they might tell you as individuals. In the group we noticed that the one who started talking seemed to set the agenda in some way. As such, we got information that we had not necessarily heard about in individual interviews. In the individual interviews they were talking without being influenced by others than the researcher.

Throughout the project the researchers kept the service user perspective in focus. And when the researcher by experience presented himself in the four focus groups, we noticed that the participants relaxed and, as in Bengtsson-Tops and Svensson (2010), they became more confident.

A study where insight in users’ experiences evolve under different circumstances by different researchers with different backgrounds might be criticised for lacking clear structures, leading to inconstancies in the information given by the participants. We are aware of this way of interpreting our method. However, our methodological choices are a way to take care of the contradictory richness of this experience and knowledge. Instead of aiming to construct a ‘master narrative’ that might be more like a biographical illusion (Bourdieu, 1998), we have opted for a method offering a possibility to gather different contradictive stories and paradoxes. As Roberts (2000) write, when arguing for a reconsideration of the significance of what has been eliminated through the adoption of principles from evidence-based medicine: ‘The clarity and simplicity of understanding that we long for can be an obstacle if such clarity is forged at the expense of denying appropriate complexity’. (p. 440).

## Analysis

We decided to use narrative analysis because it is known for its contextual complexity (Squire et al., 2013). A narrative perspective entails viewing the participants as skilful storytellers, who construct their stories about their recovery processes through narration, as we noticed that links were made between past and present and to situations, places and relationships (Clandinin et al., 2016). Inspired by Gubrium and Holstein (2009) our narrative lens is focused on the narrated places connected to what the participants said and assembled in their stories about recovery.

The first step in the analysis was to identify thematic patterns across the data set. And what struck us all was that the narrations involved many different places and people (non-human and human). We noticed that stories about recovery were stories that assembled present homes and childhood homes, difficult situations and good experiences, good people and bad people.

The second step was to analyse each narrative and sort out how each place, event and experience they spoke about was linked and evaluated in the narratives. We noticed each place, person, and situation the participants talked about and their experiences of non-human and human phenomenon (Gubrium & Holstein, 2009).

When discussing the different narratives we became aware of somewhat common features that we interpreted as typical findings across the data: (a) some stories were told chronologically, (b) some started in the present and (c) some started with a turning point. So, the last step was to construct three new narratives that represented three typical stories on recovery as assemblage. The construction of the typical stories was inspired by Malterud (2012) and what she calls artificial quotation. Artificial quotation means to condense many stories and present the main contents. She underlines that an artificial quotation maintains ‘as far as possible, the original terminology applied by the participants’. (Malterud, 2012, p. 799). She recommends writing the condensate in first-person format as a reminder to represent every participant who provided stories on this specific project. Thus, we tried to catch the significant content that we had noticed across these stories, and construct one condensed story in each category to illustrate the typical. We had two reasons for doing this. The first was to illustrate the different ways the stories were told and at the same time show the nonlinear way to recovery. The second offered the possibility to anonymise the participants’ strong personal stories that involved unfavourable characteristics of some other people.

## Results

When people with user experiences within mental health or addiction services told us about their recovery processes, it became clear that recovery stories illustrate an assemblage where humans and their environments co-exist and are interdependent. By this, assemblage challenges the idea of a master narrative of recovery or ‘a right way’ to a better life. The participants narrated their stories about recovery by also telling stories about non- recovery. Their stories covered past and present and situations, places and people (Clandinin et al., 2016). There were kinds of different relations among childhood homes, the neighbourhood, their homes of today, and places they had visited (assemblage). We constructed the first story to be told by a man and the two others by women to reflect the fact that we had more female participants in this study. And even if the genres differ, the stories highlight contradictions between the life of today and the childhood experiences and the paradoxes in the critic of the health care system at the same time as they meet helpful therapists in the same system.

## ‘The world was not a place for me’

### A chronological story by Ivar

I experienced a rough childhood, mostly because my father did not treat me, my sister, and my mother well. I think the adults in the neighbourhood must have realised that something was not right, but they seemed just not to care. Being at school was not the right place for me. There was a lot of bullying and fighting among us youths, and some of the teachers was just as bad, contributing to it rather than stop it. It was the survival of the fittest. So, I dropped out of school and tried to find a job. It was a hard time. I got deeper into alcohol and stuff. Stealing, that kind of thing. Inside I felt alone and angry, as if the world was no place for me. I got in touch with different kinds of health institutions who tried to help, but it was just not the right time.

During these years I went in and out of prison. In some way, the daily routines in prison felt safe. Small things like waking up in the morning, having breakfast in the dining room. I think it is easy to take these things for granted for ordinary people. And you get used to meeting all kinds of people there. I think this helped me. You learn to handle many different situations. I really appreciated talking with some of the staff, I came to trust them. As a matter of fact, I even consider some of them to be my friends today. I had my daughter visiting me so she could see for herself how I lived, and the staff talked to her and showed her around. They are almost like family. At the same time, you get locked in a cell.

Nowadays, if you chose to live without misusing drugs, you can have the option to serve the sentence in a halfway house if you deliver drug-clean tests. I was given this option, which was good because then I could spend more time with my daughter. It is freedom with responsibility, and it works for me.

In a way the years in and out of prison motivated me to live an ordinary life. I tried various clinics with varying results. The last clinic where I stayed at was located in the woods. At this place you work both with your mind, by participating in group therapy, and physically by working with lumber. Here, I met this man who had once been in a similar situation as myself. We talked about a lot of things, also about drugs and how to stop using them. He made me realise that I had to live with missing the comfort drugs offer me. It is like losing a good friend. This might seem odd, but drugs are like a friend for me, a friend I have had since I was 12 years old. Meeting this man made me aware of this, and this realisation made me change. It is not that I do not experience relapses; they happen from time to time and I think I will just have to live with that. The important thing is that now I see that I can manage my ups and downs. My mind set has changed. Not only because of the man I met or the stay at the clinic in the woods, but also because I take part in the municipality life where I live.

Now I even have my own apartment. I would never have even dared to hope for that just a few years ago.

## ‘I am not very well educated in terms of schooling but coming to real life I feel I am quite educated’

### A story starting today by Alice

To be at this meeting place has completely changed my life. I am so happy to meet all the people here, especially those who pop in for a coffee. We run the meeting place as an open café for everyone. This is something we have all created. We planned it, we chose colours and furniture. The atmosphere is strong. It’s like, coming through that door. ‘Hi, good to see you, you look great today’. I am bloody proud of this. You get a good energy when you are among like-minded. I feel this is my second home. People coming here cannot differentiate between those who are employed and those who are volunteers. I don’t have many tasks here, because I can’t work like others. So, in fact I am here for chatting, and some tiny, small tasks. I am not very well educated in terms of schooling but coming to real life I feel I am quite educated.

I had a difficult childhood. My home was isolated. We lived far away from other people. We couldn’t even see another house within two miles of our home. So, no one noticed what happened behind the walls. Fortunately, we moved to South of Norway. It was not that isolated, and the assaults stopped because we had neighbours close by. We could run to their house and they could help us by calling the police. My sister has meant a lot to me. She is a little bit older, she moved from our childhood home when she was 12. But before that she washed my clothes and cooked for me. And she took me to the neighbour so we could hide. He really protected us and comforted us.

At 17, I went to the GP. ‘I am not happy at home’, I said. I told him that mummy drank, but the only thing he did was to tell me to pull myself together. There was nobody. They just turned a blind eye to what was happening at home.

I stopped using drugs because of the children. I had to help myself because I have no confidence in the system. My God, I could not continue like that. Having problems like mine, abstinence, anxiety and depression follow. When I was assigned Anna as my consultant at the district psychiatric centre, then I got much more help. She had seen me for years and she was patient. Most of the therapists were fed up with trying to help us. One of them working at a service user led centre even said, ‘You are definitely the worst of them all’. But after a while I got a little flat and some money. I felt free and normal.

## ‘You realise you are better when you want to live’

### A turning point story by Sarah

‘Oh my God’, I have been everywhere meeting a lot of peculiar psychologists, until I met Olav. He is perfect. You might say he has turned me around. I lost a child. There have been a lot of tragedies in my life. I have been at clinics, hospitals and district psychiatric centres, just shit. He is just super. This never happened before I met Olav. You realise you are better when you want to live, want to go out, want to see people.

I have a lot to struggle with. And I notice, gosh, I made it without being terrified. It was fun, super fun. I feel the doctor has been more concerned with giving me medicine, than listening to me. For 5 years they gave me the highest doses of medicines, making me apathetic. They popped me full of these shitty medicines, I gained 35 kilos. I could not sleep, and they just gave me more and more and I got fatter and fatter and felt worse.

But this meeting place helps me, especially the music. We have a band. I am on drums; Peter plays bass and we have a vocalist and a guitarist. When I am too afraid to go out, the staff just pick me up; that’s great. Olav, he is a psychologist. He is fabulous.

For 10 years I had been in and out of psychiatry. The last time I was admitted, I told the staff that I deserved better. They almost laughed at me because they didn’t believe I would recover. And quickly after I came home, I suffered depression again. And I thought – no – I don’t want to be admitted again so I went to Sunset Café, a café run by the Salvation Army. I met nice people there that got me over the hump. Everyone was sitting around a table, chatting, eating. The staff sat with us. They were like us. It’s a really good place.

For years I lived in a supported housing. We had a common living room upstairs and I met others. Everyone had drug problems, so I wanted to move. It was impossible to stay free from drugs there. Later I moved, and I met Olav. But in the end, the best help I’ve had has been through a very good friend of mine. She was there for me. To me, psychiatry has been like a place where I was just stored away. They see diseases instead of people. Here at this meeting place, it’s a loving and caring family. If you want a hug, you might give a hug. So, there’s love and tenderness here, kindness.

## Discussion

### The meaning of alternation

What becomes clear when reading the stories is that a recovery story is also a story about bad experiences and a rough life. Regardless of how the stories were told, nobody told only a happy story about recovery. The participants always included experiences from a life full of challenges and problems. Helpful people they had met and good places they had visited were interwoven with people and places with different attitudes and atmospheres. In this way, life stories including narratives of recovery became an assemblage of human and non-human phenomena characterised as socio-material (Andrews & Duff, 2019), with ingredients of non-recovery experiences.

Even if the different stories seem to have a significant starting point (past, present, turning point) they all represented fluid arrangements of social and material complexity, altering between people and places. They described the connections and routes, rather than the boundaries and roots (Deleuze & Guattari, 1987), like Alice did when she talked about the route from her first isolated family home, to the next family home closer to neighbours and how this direction lead her to a place where she and her sister could hide from the assaults at home

What we find interesting is that none of the recovery stories erased the connections back in time when life had been really hard. The stories about a rough childhood or other bad experiences, became a contrast to, and a part of, their lives today. As such, the meaning of alternation between good and bad experiences became clearer. Talking about difficulties seemed to be a reminder that life was better today.

Since their hard lives had been a major part of their experiences, we can also understand alternation between non-recovery stories and recovery stories as an alternation between what is called reterritorialisation and deterritorialisation (Deleuze & Guattari, 1987). Reterritorialisation served to stabilise and maintain the ‘order’ of well-known difficult lives (Feely, 2020). Deterritorialisation illustrates a subversive process that destabilised the order and allowed for change to recovery (Feely, 2020). In many ways the well-known order for Ivar, Alice and Sarah was no order at all. Bad childhood experiences, lives with alcohol and drugs were what they were familiar with (reterritorialisation). So recovery for them was a destabilisation away from drugs, away from old friends and towards what we might call an ordinary life (deterritorialisation).

### Recovery as an assemblage

In the recovery stories it seemed important to emphasise the bad experiences as a contrast to the good ones and to alternate between reterritorialisation and deterritorialisation (Duff, 2014). Nevertheless, we will now try to go into the assemblages of what was carried out, regarding recovery related to the connection between people and places.

The starting point of a recovery story does never start with one single highly qualified professional sitting in his/her office. Even if the psychologist Olav receivedthe honour of being identified as Sarah’s turning point, she also said that ‘the best help I’ve had is through a very good friend of mine’.

A specific meeting place will not be the only place of importance for recovery, even if Ivar, Alice and Sarah all talked enthusiastically about the different meeting places they frequented. Their various experiences were based on both material and social circumstances that induced certain behaviours (DeLanda, 2006, 2016) like when Ivar was imprisoned. The prison with its strong routines, together with the prison officers that kept him under surveillance, made him feel safe, and represented a contrast to his childhood home. Alice’s childhood home was also described as a place that children should not experience, but fortunately she and her sister could escape to the neighbour’s place. So different and divergent spatial experiences mediated the routes the participants were taking and where they had come from (Trigg, 2017).

Therapeutic landscapes are about different places. Trigg (2017) stresses that places to live should be homely, and home is decidedly spatial insomuch as it serves as a point of attachment for the dweller. This was not the case for most of the participants when they grew up, because their homes and family lives threatened their safety. The ideal of the home is that it welcomes us as a place of repose, in which we take care of our self (Trigg, 2017), like the participants said about the places they lived in today. Sarah said that she ‘felt free and normal’ in her little flat.

Nevertheless, many places welcomed the participants. For Ivar, the prison became a welcoming place that organised the daily life in a predictable way. Alice described the meeting place she visited as a place she belonged to because she knew the people there and they had all planned it; and together they ran the place. The charity café Sarah spoke about was in a contradiction to what she called a ‘storage room’ (the mental hospital), and we might name such mental health institutions non-therapeutic landscapes. The participants told many stories in which psychiatric services were non-therapeutic landscapes, where they got medicine that did not help, or where they met therapists that were ‘fed up with trying to help us’, as Sarah said. But when Sarahmet Olav, who also represented the system, she had the opposite impression. Thus, assemblage perspective makes it possible for us to see the interactions between persons, their histories, and places with their characteristics and thus possibilities. To isolate just one of the parts that are present in the assemblage hinders us from fully understanding the dynamics of recovery processes. A helpful person can be present in a hindering place and contribute to the process, and vice versa. Ultimately, it will be the person who experiences recovery that gives the assemblage its meaning and role in his/her life.

Recovery seem to emerge through specific assemblages which affect people’s lives in a good way (Duff, 2016). A recovery assemblage might then be a meeting place with routes backwards to the helpful neighbours in childhood and forwards to a new home. It might be a prison or a working place in the woods. Recovery is about collecting the good stories about places and people that include the psycho-socio-material contexts (Topor et al., 2020) and at the same time remembering how bad it once was.

## Conclusion

The narratives about recovery illustrate different connections and routes that people with different challenges have described recovery as assemblages. None of these stories are similar to a well-known master narrative about recovery processes that solely focuses on positive changes within the person, giving the idea of one ‘right way’ to recovery. All the stories were assemblages with connections between social and material lives, between good experiences and bad experiences within different contexts. It seemed that the good stories became good because they were told in the light of how bad things had been. Sad stories seemed to be important contrasts and reminders to keep good experiences in mind. So, narratives about recovery are about everyday assemblages of well-being into which stories of insecurity are interwoven, without a start or stop point.

## Notes

1. ‘Experience distress, unusual experiences and troubled or troubling behavior’ is quoted from Johnstone and Boyle (2018).

2. This article is connected to a project affiliated to the research group ‘An including society’ at the University of Agder, and aims to investigate what promotes or suppresses the recovery of people with service user experiences in the field of mental health and/or addiction services.
